# Supplementary material for: Expressive rule-based modeling and fast simulation for dynamic compartments
Source: PLoS One. 2024 Oct 31;19(10):e0312813. doi: 10.1371/journal.pone.0312813 (PMC11527154; doi:10.1371/journal.pone.0312813)
Supplement: Attachment — Submitted filename: Plos Answerletter.pdf [file pone.0312813.s002.pdf]

Response to Reviewers

We thank the reviewers for their time and dedication. These detailed reviews have helped us refine our paper. We have addressed the requests and made the changes detailed below.

Reviewer 1

> *The authors addressed all of the points I raised in my previous review, and have improved the paper accordingly. I am satisfied with the revised paper.*
>
> *I have only some very minor changes to suggest.*
>
> *In their rebuttal, the authors clarified their use of the partial evaluation technique. I'd recommend adding those details in the paper as well. They already added something (around line 446) but they did not explain* in the paper *how partial evaluation is actually used.*

We added our notes from the previous response letter to the paper clarifying where partial evaluation is applied in the context of SSA.

> *I was particularly interested in the fact that structure-changing reactions are rare, yet they have a very large impact on performance since 87% of the time is spent on those. While I am sure that this figure (87%) may vary on the actual model, it clearly shows where the bottleneck is.*
>
> *If the authors know how frequently these rare reactions occur, I would recommend to add this information to the paper. In the captions of fig. 2 and 3 we can find the number of structural changes, but not their frequency (what is the total number of fired reactions?).*

We added the number of static structure reactions to the caption of Table 1 and the text. The fission yeast model executed ten dynamic and half a million static structure reactions. For the mRNA delivery model, these numbers depend on the number of lipoplexes that can unpack their mRNA into the cell. In runs where one lipoplex unpacked its mRNA into the cell, about 200 dynamic and 2 million static structure reactions occur.

> ***line 152***
> *BioNetgen -> BioNetGen (there might be other occurrences)*
>
> ***line 194***
> *"the containment is compartments" (?)*

Fixed

Reviewer 2

> *This paper addresses the modeling and the simulation of protein interaction systems with dynamical hierarchies of compartments. This topic is important because of the causal interactions between populations of proteins and their compartments, and back and forth. The simulation of these systems has been acknowledged to be scientific and technological lock, mainly for scalability issues.*
>
> *The approach combines a clever simulation architecture with some compilation optimization. Few test cases are provided (including pointers to the code).*
>
> *One key point for simulating protein interaction systems, is the dynamical update of the table of potential events. The simulation structure encapsulates such a table for each compartment. It is presented to be applied with a reaction-based simulation approach (where rules are expanded into reactions), but the approach seems quite modular and could likely be applied with a network-free approach. This choice is motivated by the assumption that most of the events will not change the hierarchy of compartments.*
>
> *To reduce the overhead of having to consider the simulation steps and the next event table for each compartment, the authors combine partial evaluation, dynamic compilation and run-time optimization.*
>
> *The paper also includes a presentation of literature.*
>
> *In my opinion, this is an important contribution to the state of the art, and it will interest the PLOS-ONE community. I thus have no doubt that it should be accepted.*

> *Nevertheless, I think that more details should be provided about compartment fission. This is a key limiting factor when dealing with dynamic compartments, especially when the content of the two sibling compartments is drawn by splitting randomly the content of the parent compartment, which would require many random number generation. Such phenomena occur when the dynamics of the compartment content are driven by the well-mixed assumption. Maybe this overhead can be avoided by using attributes, but this would deserve at least a discussion, I think.*

(see also answer below) This is not as costly in our implementaiton as it may seem. The random numbers need to be drawn only once per population, not for each specific entity. We utilize a Binomial Distribution for this. For the models realized so far, we found the random number drawing not to be a performance problem.

> *Also, focusing on the flat models (with network-free attributes potentially), the approach focuses on the model with few diversities in the molecular entities. Indeed, models with high combinatorics in protein binding cannot be modelled accurately with such approaches.*

The flat model is only an internal representation. The model is still specified using rules. Network-free attributes can be used for the high-combinatorics expressions of proteins.

> *The operational semantics of the language is only sketched. This is a good choice to explain the intuition about the approach, but some details about the models remain difficult to understand because of this (see for instance what is the meaning to multiple occurrences of the syntactic token ?c in a rhs).*

The operational semantics of ML-Rules can be found in Helms et al. 2017[1]. An explanation of using the ?c token in a fission reaction is given in the question about line 514.

[1] https://dl.acm.org/doi/10.1145/2998499

> *Please find as follows some detailed comments.*
>
> ***Line 20 3–6***
> *I would recommend including references to :*
>
> *Troels C. Damgaard, Espen Højsgaard, Jean Krivine, Formal Cellular Machinery, SASB 2012 Electronic Notes in Theoretical Computer Science, Volume 284, 2012, Pages 55-74, ISSN 1571-0661, https://doi.org/10.1016/j.entcs.2012.05.015.*
>
> *and*
>
> *Cardelli, L. (2005). Brane Calculi. In: Danos, V., Schachter, V. (eds) Computational Methods in Systems Biology. CMSB 2004. Lecture Notes in Computer Science(), vol 3082. Springer, Berlin, Heidelberg. https://doi.org/10.1007/978-3-540-25974-9_24*

Thank you for the suggestion; we included additional work on modeling compartmental dynamics (beyond Bioambients).

> ***Line 125***
> *An important method to speed up the dynamic update, consists in over-approximating the set of potential events. This is interesting when the over-approximation is less expensive to maintain. The drawback is that the validity of reactions must be checked at run-time when selected randomly.*
>
> *An example is the rectangular approximation in Kappa which considers the left hand side of rule connected-component by connected-component. (40, Section 4, Page 12)*
>
> *The dynamic updates to the table of potential events can also be optimized by exploiting the common regions among the patterns that occur in the lhs of rules.*
>
> *Boutillier, B, Ehrhard T., Krivine J. Incremental Update for Graph Rewriting. ESOP 2017*

The paper presents a method dedicated to and tuned for efficient incremental graph rewriting, which may be related to some of the non-performance-relevant aspects of our implementation. Our measurements show that the actual rewriting we perform is only a small aspect of the overall performance.

> ***Line 193-207***
> *The use of an associative and commutative operator to denote a set of concurrent entities seems to be borrowed from process calculi. (eg see Ambients, BioAmabients, Brane Calculi).*

We cited BioAmbients in the beginning but have now added further process calculi.

> ***Line 220***
> *Even worse, the distribution of the content of the sibling compartments at the fisson of their parent compartments may be drawn stochastically. (For instance, fission of a compartment under the well-mixed assumption). Each entity having a probability to be in one or the other compartment after fission. This induces a computation overhead since a random decision has to be made for each entity.*
>
> *eg see: Troels C. Damgaard, Espen Hojsgaard, Jean Krivine, Formal Cellular Machinery, SASB 2012 Electronic Notes in Theoretical Computer Science, Volume 284, 2012, Pages 55-74, ISSN 1571-0661, https://doi.org/10.1016/j.entcs.2012.05.015.*
>
> *Additional information stored in attributes, about spatial position for instance, may be used to define in which compartment each entity will be after the fission, according to the value of their attributes.*

Yes, this is also possible in ML-Rules; see language, operational semantics, and simulators described in Helms et al. 2017[2].

> ***Line 299***
> *Another example of named attributes are the counters in Kappa which support some arithmetic operations (inequality tests, incrementat, decrementat) and can be used in the rate of rules) Another example of named attributes are the counters in Kappa which support some arithmetic operations (inequality tests, increment, decrement) and can be used in the rate of rules.*
>
> *Boutillier B., Critescu I., Feret J. Counters in Kappa: Semantics, Simulation, and Static analysis. ESOP 2019*

From the beginning, the counters, arbitrary attributes, and arbitrary functions being applied to attributes and determining attributes and kinetics were one of the central features of ML-Rules (see Maus et al. 2011[3], Helms et al. 2017[2]. Counters with some other arithmetic operations are supported by many other formal modeling approaches. Therefore, the discussion of counters appears outside the scope of this paper.

> ***Line 317***
> *Discussion: The choice between network-free and flat model simulations is usually driven by the combinatorial complexity. Network-free attibutes may help, but what about binding between proteins which are central in Kappa and BNGL.*
>
> *Note also that Kappa counters could be considered as 'network-free-integers'.*

Explicit bindings are supported traditionally by ML-Rules by generating new unique names. This may be computationally inefficient; therefore, in Helms et al., we presented a specific simulator working on a constrained class of ML-Rule models where variables denote bindings between two entities. Another option that we currently apply in developing a large ML-Rules model with complex signalosomes is to express this with attributes and dynamic compartments by collecting entities in a compartment instead of linking them with a binding site.

> ***Line 333:***
> *A reference to unit/dimension analysis could be provided, such as:*
>
> *Generalized homogeneous polynomials for efficient template-based nonlinear invariant synthesis. Kensuke Kojima, Minoru Kinoshita1, and Kohei Suenaga. In: Static Analysis Symposium (SAS 2016).*

Thank you for the suggestion; we added the reference.

> ***Line 351.***
> *The approach seems to me to be more modular than described in the presentation. It could likely be applied to network free, flat representation, and hybrid solutions between them, depending on the nature of the model.*
>
> *Compartment fission has been identified to be costly, since the distribution of the content must be drawn.*

[2]https://dl.acm.org/doi/10.1145/2998499
[3]https://bmcsystbiol.biomedcentral.com/articles/10.1186/1752-0509-5-166

This is not as costly in our implementation as it may seem. The random numbers need to be drawn only once per population, not for each specific entity. We utilize a Binomial Distribution for this. For the models realized so far, we found the random number drawing not to be a performance problem.

> ***Line 514***
> *Is the content of a cell is split when the cell is divided, or duplicated?*
>
> *In the source code, the syntactic token ?c appears twice in the rhs. What does it mean? Is the content of the place-holder duplicated?*

The cell content can be divided or duplicated in a split event. In the specific fission yeast model used in the ML-Rules2 paper, the content is duplicated, and therefore, the same is done in the ML-Rules3 model (realized by using ?c two times on the RHS), so to replicate the results. If the content of a compartment needs to be divided (symmetrical or asymmetrical), this can be done with the rule

$$\text{Mito(?m1}[R_1]\text{+?m2}[R_2]\text{)} \rightarrow \text{Mito(?m1) + Mito(?m1)}$$

where R_1 and R_2 specify at which ratio the content of the Mito should be split into ?m1 and ?m2.

Additional Comments

> *When submitting your revision, we need you to address these additional requirements.*
>
> *Please ensure that your manuscript meets PLOS ONE's style requirements, including those for file naming. The PLOS ONE style templates can be found at https://journals.plos.org/plosone/s/file?id=wjVg/PLOSOne_formatting_sample_main_body.pdf and https://journals.plos.org/plosone/s/file?id=ba62/PLOSOne_formatting_sample_title_authors_affiliations.pdf*
>
> *Thank you for stating the following in the Acknowledgments Section of your manuscript: Figures 1 was created with BioRender.com. This work was funded by the Deutsche Forschungsgemeinschaft (DFG, German Research Foundation) SFB 1270 – 299150580 ELAINE and grant 225222086.*
>
> *We note that you have provided funding information that is not currently declared in your Funding Statement. However, funding information should not appear in the Acknowledgments section or other areas of your manuscript. We will only publish funding information present in the Funding Statement section of the online submission form. Please remove any funding-related text from the manuscript and let us know how you would like to update your Funding Statement. Currently, your Funding Statement reads as follows: P.H. received frunding from the Deutsche Forschungsgemeinschaft (DFG, German Research Foundation, https://www.dfg.de/en) SFB 1270 – 299150580 ELAINE. T.K. received frunding from the Deutsche Forschungsgemeinschaft (DFG, German Research Foundation, https://www.dfg.de/en) grant 225222086 The DFG did not play a role in the study design, data collection and analysis, decision to publish, or preparation of the manuscript.*
>
> *Please include your amended statements within your cover letter; we will change the online submission form on your behalf.*

We removed the mention of the funding from the acknowledgment section. The funding statement, as stated above, is correct content-wise but contains a typo (frunding → funding).

> *Please review your reference list to ensure that it is complete and correct. If you have cited papers that have been retracted, please include the rationale for doing so in the manuscript text, or remove these references and replace them with relevant current references. Any changes to the reference list should be mentioned in the rebuttal letter that accompanies your revised manuscript. If you need to cite a retracted article, indicate the article's retracted status in the References list and also include a citation and full reference for the retraction notice.*
